# Comparison on Surgical Outcomes of Mini-Versus Standard-Percutaneous Nephrolithotomy in Staghorn Calculi: A Systematic Review and Meta-Analysis

**DOI:** 10.5152/tud.2025.24125

**Published:** 2025-03-06

**Authors:** Noka Yogahutama, Muhammad Isa Fuad Affan, Alan Primi Ladese, Nicholas Abraham

**Affiliations:** 1 Mitra Sehat Hospital, Sleman, Indonesia; 2 Department of Urology, Dr. R. Soetrasno Regional General Hospital, Rembang, Indonesia; 3 Dr. R. Soetrasno Regional General Hospital, Rembang, Indonesia; 4 Immanuel Hospital, Bandung, Indonesia

**Keywords:** Mini percutaneous nephrolithotomy, minimally invasive percutaneous nephrolithotomy, nephrolithiasis, nephrolithotomy, percutaneous, Staghorn calculi, Staghorn stone

## Abstract

Percutaneous Nephrolithotomy (PCNL) has become the standard for managing staghorn calculi. Smaller access sheath Mini-PCNL (M-PCNL) has been known for its advantages in surgical outcomes compared with Standard-PCNL (S-PCNL) in various settings. We conducted this systematic review to compare surgical outcomes and postoperative complications of M-PCNL vs. S-PCNL in staghorn calculi treatment. A systematic search of the literature was performed on PubMed, Cochrane Library, ProQuest, Scopus, ClinicalTrials.gov, and Google Scholar according to the Preferred Reporting Items for Systematic Reviews and Meta-Analyses Statement (PRISMA Statement). Five randomized controlled trial (RCT) and 5 cohort studies were included. Risk of bias assessment was evaluated using Cochrane risk of bias (RoB) 2 and Cochrane RoB in Non-randomized Studies – of Interventions (ROBINS-I). Ten studies involving 1733 staghorn calculi patients met the inclusion criteria. The stone-free rate (SFR) was comparable (odds ratio (OR) 1.13, 95% CI: 0.90-1.43, *P* = .28) compared to S-PCNL. Pooled analysis showed that M-PCNL resulted in a significantly lower shorter operative time (mean differences (MD) 14.06, 95% CI: 6.09-22.03, *P* < .001), lower blood transfusion (OR 0.46, 95% CI: 0.29-0.72, *P* < .001), and lower hemoglobin drop (MD −0.37, 95% CI: −0.72 to −0.03, *P* = .03) compared to S-PCNL. This meta-analysis suggests that while M-PCNL has comparable SFR to S-PCNL, it offers benefits in terms of shorter operative time, reduced blood transfusion needs, and less hemoglobin drop.

**Main Points**

- The first line treatment of staghorn calculi is percutaneous nephrolithotomy (PCNL).
- Mini-PCNL (M-PCNL) can be an effective option for standard PCNL for staghorn calculi management.
- Mini-PCNL is strongly associated with less bleeding compared to standard PCNL for staghorn calculi management.
- Longer operative time and an increased risk of postoperative fever must be considered before performing M-PCNL in staghorn calculi patients.

## Introduction

Currently, percutaneous nephrolithotomy (PCNL) has become the standard of care for renal calculi >20 mm and is also recommended by the American Urological Association (AUA) and the European Urological Association (EAU) as the first-line treatment for staghorn calculi.^1,2^ Since its introduction by Fernström and Johanson in 1976, PCNL has been widely used and has become common among urologists.^3^ This invasive procedure has a notable rate of complications for staghorn calculi, with one of the most serious being extensive renal bleeding requiring blood transfusion.^4,5^ This complication is often caused by the large tract size and the need for multiple tracts.^5^

To reduce the complication rate associated with traditional PCNL, smaller “miniaturized” PCNL access sheaths have been used since their introduction in 1998 for children.^6^ The use of miniaturized PCNL has been extended to adults, showing fewer complications, particularly less blood loss. As Standard-PCNL (S-PCNL) became the first-line treatment for staghorn calculi, the use of M-PCNL has increased. Mini-PCNL (M-PCNL) is defined as using instrument sizes <24 Fr, whereas S-PCNL uses instrument sizes between 24 and 30 Fr.^7^ A meta-analysis by Qin et al^8^ considered M-PCNL an effective and reliable alternative to S-PCNL for renal calculi larger than 2 cm, with comparable stone free rate (SFR) and fewer complications. Staghorn calculi, which are complex branched renal calculi, present a special challenge for treatment with PCNL. The use of M-PCNL instead of S-PCNL for treating staghorn calculi remains a debated topic concerning its complexity in achieving SFR and postoperative complications.

Several systematic reviews and meta-analyses comparing M-PCNL and S-PCNL have shown no significant difference in SFR between the 2 methods.^8-12^ However, none of these meta-analyses specifically compared M-PCNL and S-PCNL for patients with staghorn calculi. Therefore, we conducted a systematic review and meta-analysis to compare surgical outcomes and postoperative complications of each procedure in treating staghorn calculi.

## Materials and Methods

### Data Sources and Search Strategies

This systematic review and meta-analysis was registered in the PROSPERO database (CRD42024571234). Several changes were made in this review compared to the registered protocol. The present study followed the 2020 Preferred Reporting Items for Systematic Reviews and Meta-Analyses (PRISMA) guidelines.^13^ We included a randomized controlled trial (RCT) and prospective or retrospective cohort studies evaluating patients with staghorn calculi who were managed with either M-PCNL or S-PCNL up to August 2024. We systematically searched for studies in various online databases, including PubMed, Scopus, ProQuest, Cochrane Central Register of Controlled Trials (CENTRAL), and ClinicalTrials.gov. Additionally, we included studies from gray literature, such as Google Scholar. Medical Subject Headings (MeSH) terms and keywords were used in the literature search, as shown in Supplementary Table 1.

### Inclusion Criteria and Exclusion Criteria

The inclusion criteria for this systematic review were determined using the PICOS framework, which consists of population (P), intervention (I), comparison (C), outcome (O), and study design (S). According to this framework, the criteria were as follows: (P): patients diagnosed with staghorn calculi; (I): Mini-PCNL (M-PCNL) with an access sheath size <24 Fr; (C): Standard-PCNL (S-PCNL) with an access sheath size ≥24 Fr; (O): Surgical outcomes and postoperative complications; and (S): RCT and prospective or retrospective cohort studies. We excluded studies that were case reports, conference abstracts, reviews, meta-analyses, duplicate studies, editorials, or not available in English language. We included only studies that reported at least the SFR, as this was the primary surgical outcome.

### Data Extraction and Study Quality

Data extraction from the included studies was performed independently by 3 investigators (N.Y, A.P.L, N.A.) and then recorded and cross-checked using Microsoft Excel version 15.20 (Microsoft Corporation, Washington, USA, 2016). Several pieces of information were extracted, including author, study origin, study period, study design, number of cases, mean age, mean body mass index (BMI), gender, stone size or stone score, position, access sheath size, nephroscope size, dilation technique, and lithotripsy technique. Additional information necessary for meta-analysis calculations was also extracted, including SFR, number of tract accesses, length of operative time, number of blood transfusions, postoperative hemoglobin drop, number of postoperative fever cases, number of cases with any complications, and length of hospital stay. Quality assessment was performed by 3 investigators (N.Y, A.P.L, N.A.) using the recommended tools in the Cochrane Handbook for Systematic Reviews of Interventions.^14^ The quality of RCT studies was assessed using the Cochrane RoB version 2.0 (RoB 2.0) across 5 domains: randomization process, deviations from intended interventions, missing outcome data, measurement of the outcome, and selection of the reported result. Overall RoB was classified as low, some concerns, or high. Cohort studies were assessed using the RoB in nn-randomized studies—of Interventions (ROBINS-I) tool across 7 domains: confounding, selection of participants, classification of interventions, deviations from intended interventions, missing data, measurement of outcomes, and selection of the reported result. Overall RoB was classified as low, moderate, serious, or critical. In cases of disagreement, a second investigator (M.I.F.A.) was consulted to help resolve the issue through discussion.

### Statistical Analysis

Meta-analyses were performed when 2 or more studies reported the same outcomes. Dichotomous data were presented as odds ratios (ORs) with 95% CI, while continuous data were presented as mean differences (MD). For continuous data presented as median and range values, we used the formulas described by Hozo et al^15^ to determine the mean and standard deviation (SD). An OR greater than 1 indicates increased chances of surgical outcome success rates and increased chances of postoperative complications in M-PCNL studies. All results were pooled using the Cochran–Mantel–Haenszel method. A *P*-value (*P*) of less than .05 was considered statistically significant. Heterogeneity between included studies was assessed using the *I*² value. According to the Cochrane Handbook for Systematic Reviews of Interventions, *I*² values of <30%, 30%-60%, 50%-90%, and 75%-100% represent low, moderate, substantial, and considerable heterogeneity, respectively.^14^ If the *I*² value was >50%, the heterogeneity was considered statistically high, and a random-effects model was used. If the *I*² value was ≤50%, the heterogeneity was considered statistically low, and a fixed-effect model was used. The random-effects model was also used for subgroup analysis, as recommended in the Cochrane Handbook for Systematic Reviews of Interventions.^14^ Forest plots were generated and interpreted accordingly. Funnel plots were generated to assess asymmetry, which may indicate publication bias in the included studies. All meta-analyses were conducted using Review Manager (RevMan) version 5.4 software (The Cochrane Collaboration, United Kingdom, 2020). Sensitivity analysis was performed for each study to assess the stability of the results and the influence of individual studies by sequentially removing each study one at a time. Results from the statistical and sensitivity analyses were recorded in Microsoft Excel version 15.20.

## Results

### Search Result and Study Characteristics

**Figure 1.**
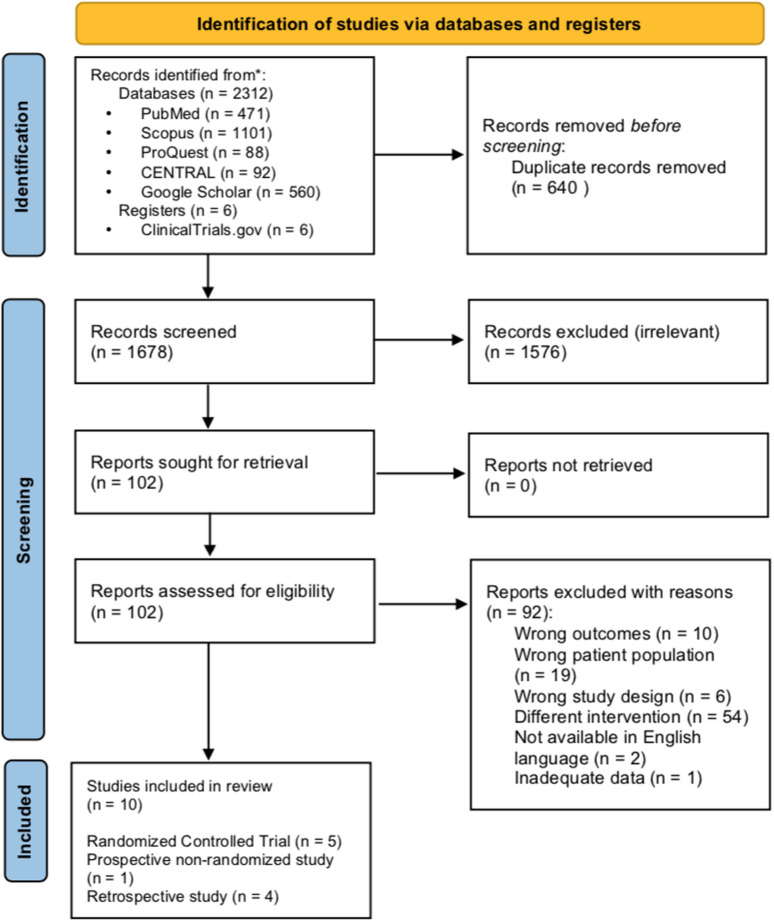
Literature search and screening for eligible studies based on the PRISMA 2020 flowchart.

The literature search and study screening for this review followed the 2020 PRISMA flow chart guidelines,^13^ as shown in Figure 1. The search retrieved 2312 papers, of which 640 duplicates were removed. Of the remaining 1678 papers, 1576 were excluded after reviewing the titles and abstracts. The full texts of the remaining 102 papers were examined, and 92 were excluded as their content was irrelevant to this study. Finally, 10 studies, including 5 RCT, 4 retrospective cohort studies, and 1 prospective non-randomized cohort study, were included for meta-analysis. A total of 1733 patients diagnosed with staghorn calculi were included in these 10 studies, comprising 926 patients who underwent M-PCNL and 807 patients who underwent S-PCNL. A summary of the included comparative studies is presented in Table 1, and the baseline characteristics of these studies are detailed in Table 2. The types of staghorn calculi in the patient population varied from partial to complete staghorn calculi, with one study by Zhong et al^17^ including borderline staghorn calculi. The size of access sheaths ranged from 16 Fr to 20 Fr in the M-PCNL group and from 24 Fr to 30 Fr in the S-PCNL group. The lithotripsy and dilation techniques also varied, as described in Table 2. The definition of SFR varied among the included studies, ranging from complete clearance of stone fragments to residual fragments of ≥3-≥5 mm.

### Risk of Bias Assessment

**Supplementary Figure 1.**
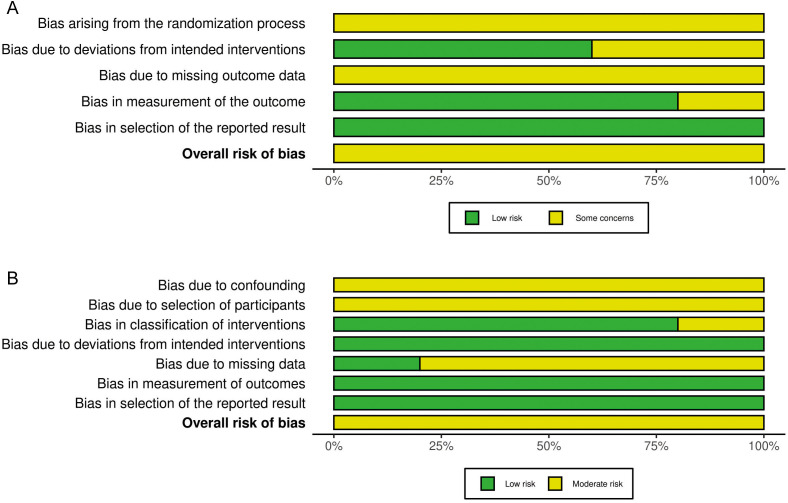
a, b. Risk of bias assessment of included studies. (a) RoB 2.0 for RCT studies, (b) ROBINS-I for included cohort studies.

The overall RoB in the 5 RCT studies, assessed using RoB 2.0, were classified as “some concerns,” as shown in Supplementary Figure 1. All 5 RCT studies were evaluated and found to have concerns related to the randomization process, as blinding between patients and practitioners was nearly impossible in the surgical context. No bias arising from the selection of reported results was observed. The 5 cohort studies were assessed using ROBINS-I and classified as having a “moderate” overall RoB, as shown in Supplementary Figure 1. Bias in these cohort studies was attributed to confounding, selection bias, and missing data due to the observational nature of the included studies.

### Publication Bias and Sensitivity Analysis

**Supplementary Figure 2.**
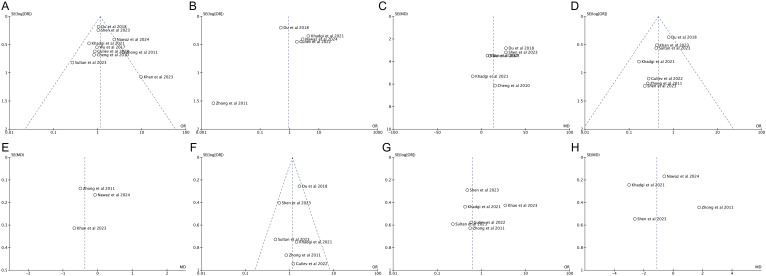
a-h. Funnel plot of meta-analysis reporting surgical outcomes and postoperative complications. (a) SFR, (b) single tract access, (c) operative time, (d) blood transfusion, (e) hemoglobin drop, (f) postoperative fever, (g) overall postoperative complications, (h) hospital stay.

Funnel plots were generated for each meta-analysis, and sensitivity analysis was conducted using the “leave-one-out” method. Asymmetry was found only in the blood transfusion meta-analysis group as shown in Supplementary Figure 2, which was attributed to the homogeneity of the included studies and their similar results. Also, sensitivity analysis of this meta-analysis group showed similar results when each study was excluded as shown in Supplementary Table 5. In the single tract access meta-analysis group as shown in Supplementary Table 3, a study by Zhong et al^17^ was identified as a significant outlier that affected the pooled result. This was due to the study’s inclusion criteria, which only encompassed M-PCNL with multiple tract access. Additionally, the study by Du et al^19^ also influenced the pooled result in this meta-analysis group, likely because of the large differences in population size compared to other studies included. Similarly, the pooled result in the postoperative fever meta-analysis group was also altered by these studies as shown in Supplementary Table 7.

### Surgical Outcomes

#### Stone Free Rate and Subgroup Analysis:

**Figure 2.**
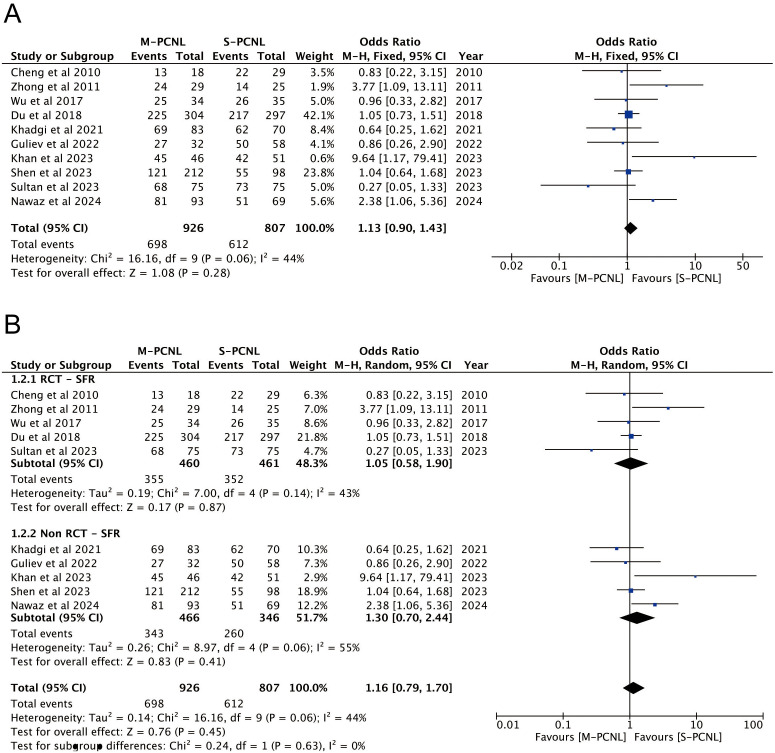
Forest plot for (A) SFR, and (B) SFR subgroup analysis based on studies design (RCT and non-RCT) between the groups.

Ten studies^16-25^ reported the SFR with 926 patients underwent M-PCNL and 807 patients underwent S-PCNL. The total SFRs were 75.3% (698/926) for M-PCNL and 75.8% (612/807) for S-PCNL. The analysis showed no significant difference in SFR between M-PCNL and S-PCNL (OR 1.13, 95% CI: 0.90-1.43, *P* = .28) as shown in Figure 2. However, there was moderate heterogeneity in this meta-analysis group (*I*² = 44%). Further subgroup analysis included 5 studies reporting SFR for both RCT^16-19,24^ and non-RCT^20-23,25^ subgroups. In the RCT studies subgroup, 355 patients underwent M-PCNL and 352 underwent S-PCNL, with a total SFR of 77.1% (355/460) and 76.3% (352/461), respectively. In the non-RCT studies subgroup, 466 patients underwent M-PCNL and 346 underwent S-PCNL, with a total SFR of 73.6% (698/926) and 75.1% (612/807), respectively. The pooled OR indicated no significant differences in SFR between the RCT and non-RCT subgroups (OR 1.05, 95% CI: 0.58-1.90, *P* = .87 vs. OR 1.30, 95% CI: 0.70-2.44, *P* = .41) as shown in Figure 2, with moderate heterogeneity observed in both subgroups (*I*² = 43% vs. *I*² = 55%).There was no observed heterogeneity in terms of effect sizes between subgroups (*I*² = 0%), indicating no variability of outcomes between the subgroups.

#### Single Tract Access:

**Figure 3.**
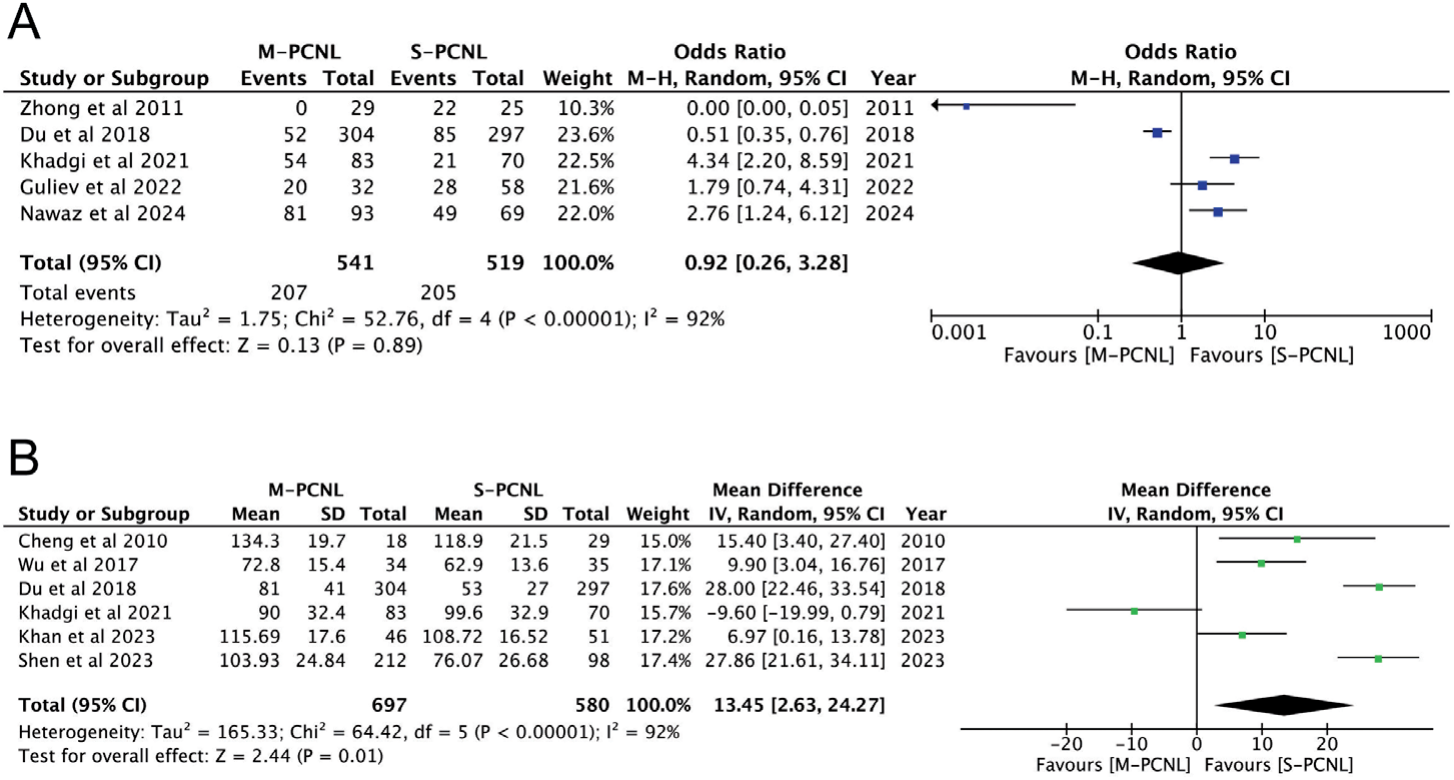
Forest plot for (A) single tract access and (B) operative time between the groups.

Five studies^17-21,25^ reported that 207 out of 541 patients underwent M-PCNL and 205 out of 519 patients underwent S-PCNL with single tract access. The analysis showed no significant difference between M-PCNL and S-PCNL regarding the achievement of single tract access (OR 0.92, 95% CI: 0.26-3.28, *P* = .89) as shown in Figure 3. A considerable heterogeneity was observed in this meta-analysis group (*I*² = 92%).

#### Operative Time:

Seven studies^16-20,22,23^ reported on operative time for 726 patients who underwent M-PCNL and 605 patients who underwent S-PCNL. Mini-PCNL (M-PCNL) was associated with a statistically significant longer operative time compared to S-PCNL (MD 14.06, 95% CI: 6.09-22.03, *P* = < .001) as shown in Figure 3. However, there was considerable heterogeneity in this meta-analysis group (*I*² = 91%).

### Postoperative Complications

#### Blood Transfusion:

**Figure 4.**
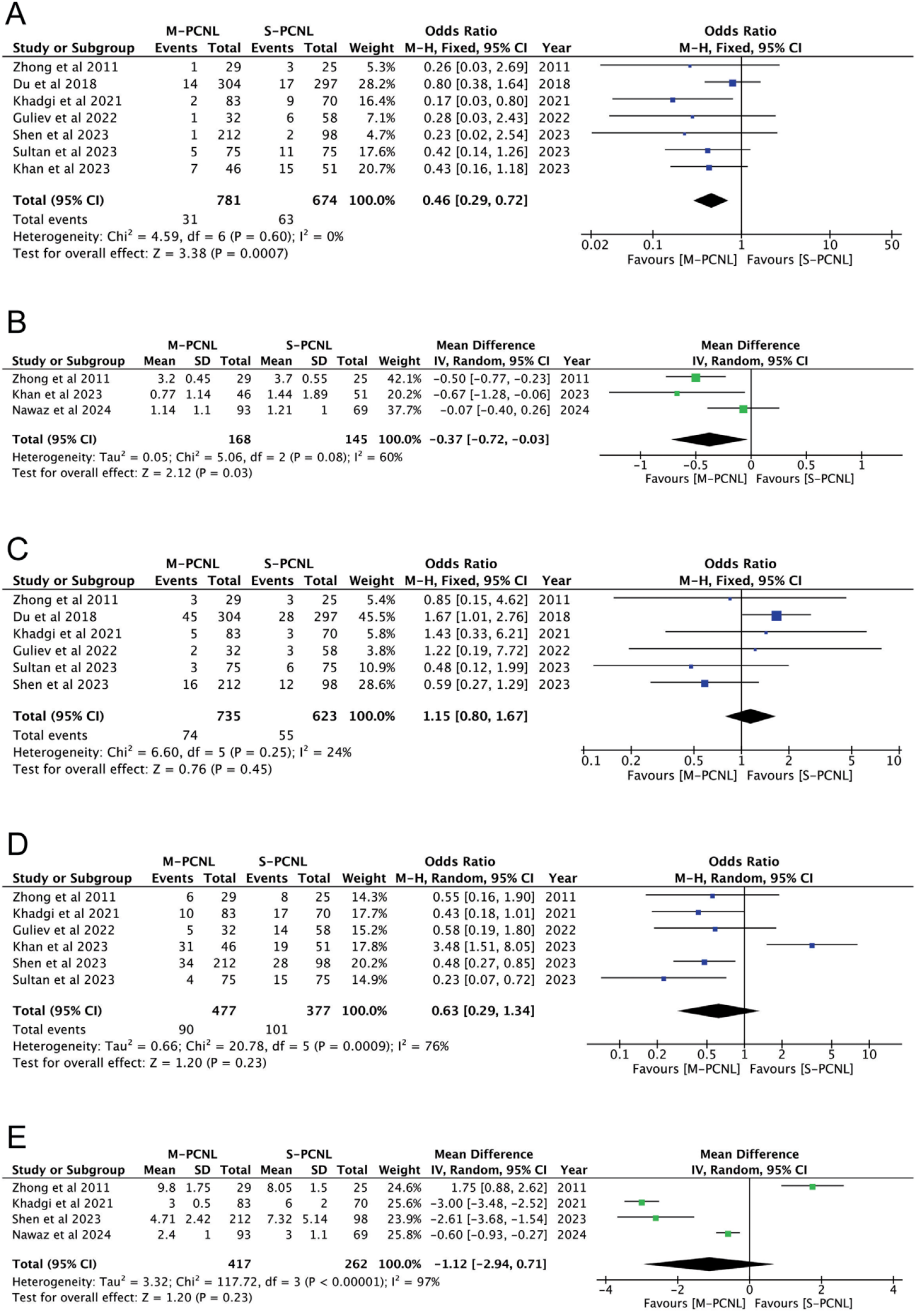
Forest plot for (A) blood transfusion, (B) hemoglobin drop, (C) postoperative fever, (D) overall postoperative complications, and (E) hospital stay between the groups.

Seven studies^17,19-24^ reported on blood transfusion requirements for 31 out of 781 patients underwent M-PCNL and 63 out of 674 patients underwent S-PCNL. Mini-PCNL (M-PCNL) was associated with a statistically significant lower risk for blood transfusion compared to S-PCNL (OR 0.46, 95% CI: 0.29-0.72, *P* < .001) as shown in Figure 4. There was low heterogeneity in this meta-analysis group (*I*² = 0%).

#### Hemoglobin Drop:

Three studies^17,22,25^ reported hemoglobin drop for 168 patients who underwent M-PCNL and 145 patients who underwent S-PCNL. In this meta-analysis group, M-PCNL was associated with a statistically significant lower hemoglobin drop compared to S-PCNL (MD −0.37, 95% CI: −0.72 to −0.03, *P* = .03) as shown in Figure 4. However, the heterogeneity in this meta-analysis group was considered substantial to moderate (*I*² = 60%).

#### Postoperative Fever:

Six studies^17,19-21,23 24^ reported on postoperative fever, with 74 out of 735 patients underwent M-PCNL and 55 out of 623 patients underwent S-PCNL experiencing postoperative fever. Mini-PCNL was associated with a statistically insignificant, slightly higher risk of postoperative fever compared with S-PCNL (OR 1.15, 95% CI: 0.80-1.67, *P* = .45) as shown in Figure 4. The heterogeneity in this group analysis was considered low (*I*² = 24%).

#### Overall Postoperative Complications:

Six studies^17,20-24^ reported on postoperative complications, with 90 out of 477 patients underwent M-PCNL and 101 out of 377 patients underwent S-PCNL experiencing at least 1 postoperative complication. Mini-PCNL was associated with a lower risk of experiencing any postoperative complications compared to S-PCNL (OR 0.63, 95% CI: 0.29-1.34, *P* = .23). The heterogeneity in this meta-analysis group was considered substantial (*I*² = 76%).

#### Hospital Stay:

Four studies^17,20,23,25^ reported on hospital stay length for 417 patients who underwent M-PCNL and 262 patients who underwent S-PCNL. Mini-PCNL was associated with a statistically insignificant shorter hospital stay compared to S-PCNL (MD −1.12, 95% CI: −2.94 to 0.71, *P* = .23). However, there was considerable heterogeneity in this meta-analysis group (*I*² = 97%).

## Discussion

Staghorn calculi are complex renal calculi that occupy the renal pelvis and at least one renal calyx, most commonly composed of struvite (magnesium ammonium phosphate), which is associated with urinary tract infections (UTI).^26^ The treatment of staghorn calculi is challenging due to their shape and impact on renal function. Advances in technology have established PCNL as the first-line treatment for staghorn calculi, as recommended by the EAU and AUA.^1,2^ Although PCNL is considered effective and safe, its success heavily depends on the operator’s skill and experience, and it is associated with a higher rate of complications, including intraoperative bleeding and postoperative infections, compared to non-staghorn calculi.^27,28^ These complications are thought to result from the large tract size and multiple tracts required in PCNL.^4 5^ Efforts are currently being made to reduce PCNL complications while maintaining effectiveness by using M-PCNL with a smaller access sheath diameter (<24 Fr) compared to S-PCNL with a larger sheath diameter (24-30 Fr).^7^ According to 5 meta-analyses, M-PCNL is reported to have comparable safety and effectiveness to S-PCNL for treating renal calculi,^8-12^ including studies by Qin et al,^8^ which reported similar findings for stones larger than 2 cm. However, there is no definitive conclusion on the safety and efficacy of M-PCNL specifically for staghorn calculi. Currently, RCT and cohort studies comparing M-PCNL and S-PCNL for staghorn calculi are limited. To our knowledge, this meta-analysis is the first to evaluate the safety and efficacy of M-PCNL compared to S-PCNL in patients with staghorn calculi. The aim of this study was to assess and compare the surgical outcomes and postoperative complications of M-PCNL and S-PCNL for treating staghorn calculi.

The total SFR for M-PCNL and S-PCNL in this study was very similar, around 75%. This result is lower compared to the approximately 86% SFR reported in a review by Deng et al,^12^ which included all types of renal calculi patients. The lower total SFR in our study can be attributed to the complex nature of staghorn calculi. Additionally, the total SFR in this study was consistent across subgroup analyses of RCT and non-RCT studies. Specifically, the total SFR for M-PCNL and S-PCNL in non RCT studies was slightly lower compared to that in RCT studies (73.6% and 75.1% vs. 77.1% and 76.3%). The SFR in this study was higher than the 56.9% reported by the Clinical Research Office of the Endourological Society for staghorn calculi patients.^29^ Statistical analysis revealed no significant difference in SFR between M-PCNL and S-PCNL, which aligns with previous meta-analyses that included patients with non staghorn calculi.^8-12^ However, in subgroup analyses, M-PCNL demonstrated a slightly better SFR compared to S-PCNL in both RCT and non-RCT studies. This contrasts with Deng et al,^12^ which found S-PCNL to have a statistically significant advantage in SFR in non-RCT studies, although RCT studies in the same research reported no significant difference in SFR between M-PCNL and S-PCNL. Several factors that may influence the surgical outcomes in our study are worth acknowledging. These include baseline characteristics such as BMI, staghorn calculi size and type, access sheath and nephroscope size, and lithotripsy technique. Additionally, the definition of SFR varied among the included studies due to the lack of a standardized SFR definition. The postoperative follow-up time also affects SFR, as longer follow up generally results in higher SFR due to additional time for flushing out calculi fragments through urine.^11^ Furthermore, the method used to assess SFR varied among the studies, with most employing X-ray (KUB) and CT scans as needed. This variation could lead to false-negative SFR results, as CT scans are considered the most accurate method for assessing SFR.^11^ Despite these factors, our study suggests that M-PCNL is an effective alternative to S-PCNL for treating staghorn calculi in terms of SFR.

The number of tracts used in PCNL also influences the SFR results. Theoretically, multiple tracts are often required in a single PCNL session for staghorn calculi to achieve an SFR status due to their complexity and shape.^30,31^ In this study, M-PCNL showed a comparable single-tract access to achieve SFR in both M-PCNL and S-PCNL groups. However, the number of tracts used is primarily based on the endourologists’ experience and preference, as they are most familiar with the renal anatomy and the surgical procedure, considering the additional complications associated with multiple tracts compared to S-PCNL.^32^

As expected, this study revealed a statistically significant longer operative time for M-PCNL compared to S-PCNL in the treatment of staghorn calculi, consistent with previous meta-analyses.^8-12^ Previous meta-analyses generally found M-PCNL to be less than 10 minutes longer than S-PCNL. In this study, M-PCNL had an operative time 14.06 minutes longer than S-PCNL. A subgroup analysis by Jiao et al^11^ reported a 20.83 minute longer operative time for M-PCNL compared to S-PCNL specifically for staghorn calculi, based on only 2 RCT studies. This suggests that M-PCNL may result in a notably longer operative time for staghorn calculi due to their complexity. Endourologists should consider this extended operative time when planning M-PCNL for staghorn calculi patients. The longer operative time is also likely influenced by other factors, including the typically poorer visual field with M-PCNL and the need to fragment calculi into smaller pieces to pass through the narrower tract.^8^ Additionally, the higher number of percutaneous tracts used in M-PCNL also contributes to the increased operative time compared to S-PCNL. This is supported by Deng et al,^12^ which noted that larger tract sizes are associated with shorter operative times.

One of the most critical factors related to PCNL is bleeding-related complications, as staghorn calculi have been linked to significant bleeding during the procedure.^26,33^ Significant bleeding can be influenced by factors such as operative time, stone size, sheath size, number of tracts, and the volume of cases.^5,33,34^ Bleeding during PCNL is often associated with puncture injuries, which can be exacerbated by larger access sheaths that damage renal blood vessels and surrounding organs.^35^ Furthermore, the complex nature of staghorn calculi, involving multiple renal calyces, often requires multiple tracts, which can further exacerbate bleeding.^30,31^ In this study, the need for blood transfusions was significantly lower in M-PCNL compared to S-PCNL, likely due to the smaller sheath size that reduced the bleeding. This finding aligns with previous meta-analyses that reported significantly lower need of blood transfusion with M-PCNL.^8,10-12^ Additionally, the analysis of hemoglobin drop in this study also confirmed these results, with M-PCNL associated with a lower hemoglobin drop compared to S-PCNL, as revealed by previous meta-analyses.^8-11^ Despite the reduced risk of blood transfusion and hemoglobin drop with M-PCNL, endourologists must consider the staghorn calculi’s shape and size, which may lead to extended operative times due to multiple tracts, poorer visual fields, and the need to fragment calculi into smaller pieces.^8^

In this study, postoperative fever was slightly higher in the M-PCNL compared to the S-PCNL, although this difference was not statistically significant. This finding is consistent with previous meta-analyses, which also reported statistically insignificant differences,^8,10,11^ though some previous meta-analyses have reported the opposite.^9,12^ Theoretically, the smaller tract diameter of M-PCNL could increase intra renal pressure (IRP) that led to the risk of bacterial seeding.^7^ In addition, the struvite composition of staghorn calculi, which is associated with UTI, further heightens the risk of postoperative fever.^26^ Despite this, overall postoperative complications were slightly lower in the M-PCNL group compared to S-PCNL. Furthermore, the M-PCNL showed a slightly shorter hospital stay compared to the S-PCNL in this study. Previous meta-analyses have also reported similar results with significant findings.^8-11^ Shorter hospitalizations in the M-PCNL may be attributed to fewer complications, though a detailed analysis of complications was limited in this study due to insufficient data. Nonetheless, the lower complication rate and shorter hospitalization period are positive factors supporting the use of M-PCNL for staghorn calculi.

As the technology of percutaneous surgery continues to develop further, consideration of newer tools and techniques should help endourologists improve the efficacy and safety of their PCNL procedures. Not only the trends to “*miniaturized the PCNL*” as mentioned by Lahme et al,^36^ but the innovation and development of PCNL armamentarium and techniques are evolving, changing strategies for lithotripsy and stone removal.^37^ One of the important innovation is the addition of suction to PCNL, which is available in the sheath, the scope, or the energy device with advantages of reduction in infectious complications and enhancement of SFR.^38^ Even the low IRP can be maintained with the vacuum effect of suction in M-PCNL.^38^ The newer lithotripters such as Holmium: Yttrium-Aluminum-Garnet (Ho:YAG) has been widely accepted as the gold standard for M-PCNL.^39^ Even, high power Ho:YAG lasers are also being applied in M-PCNL.^39^ Currently, the application of high-power lasers, stone dust evacuation techniques, new suction-integrated nephrostomy access sheaths, and handpieces is changing and evolving the way percutaneous surgery is performed.

This review acknowledges several limitations. Unlike other studies that compare M-PCNL and S-PCNL, our study focuses exclusively on staghorn calculi rather than general renal calculi.^8-12^ Additionally, the inclusion of both RCT and cohort studies may lower the overall evidence level. Most of the included studies exhibit “some concerns” bias, primarily due to randomization issues and the difficulty of blinding in surgical contexts. Differences in baseline characteristics among studies such as BMI, stone size, access sheath size, nephroscope size, dilatation technique, lithotripsy technique, SFR definitions, and follow up methods could potentially confound the surgical outcomes. We also consider that these differences may contribute to the wide heterogeneity in our study. A publication bias was also noted in the meta-analysis of blood transfusion; however, sensitivity analysis showed no deviation from previous studies. Despite these limitations, this review provides the most current evidence on M-PCNL versus S-PCNL specifically for staghorn calculi patients.

In this systematic review and meta-analysis, the authors conclude that M-PCNL is an effective alternative to S-PCNL for treating staghorn calculi. Despite the inherent complexity of staghorn calculi, M-PCNL shows comparable SFR, shorter hospital stays, lower need for blood transfusions, lower hemoglobin drop, and fewer overall postoperative complications compared to S-PCNL. However, considerations for longer operative time and the risk of postoperative fever are important. Further research with larger, well-designed RCT is needed to confirm these findings.

## Supplementary Materials

Supplementary Material

## Figures and Tables

**Table 1.** Summary of Included Comparative Studies

Study	Origin of Study	Study Period	Study Design	Cases, n		References
				M-PCNL	S-PCNL	
Cheng et al, 2010	China	2004-2007	RCT	18	29	(16)
Zhong et al, 2011	China	2008-2009	RCT	29	25	(17)
Wu et al, 2017	China	2014-2015	RCT	34	35	(18)
Du et al, 2018	China	2009-2014	RCT	304	297	(19)
Khadgi et al, 2021	Nepal	2015-2019	Cohort retrospective	83	70	(20)
Guliev et al, 2022	Russia	NI	Cohort retrospective	32	58	(21)
Khan et al, 2023	India	2019-2022	Cohort retrospective	46	51	(22)
Shen et al, 2023	China	2019-2022	Cohort retrospective	212	98	(23)
Sultan et al, 2023	Pakistan	2017-2019	RCT	75	75	(24)
Nawaz et al, 2024	Pakistan	2020-2023	Cohort prospective non randomized	93	69	(25)

n, amount; RCT, randomized controlled trial.

**Table 2.** Baseline Characteristics of Included Comparative Studies

Study	Mean Age		Mean BMI		Gender (M/F)		Stone Size/Stone Score		Position	Access Sheath Size		Nephroscope Size		Dilatation Technique		Lithotripsy Technique		SFR Definition	SFR Follow Up
	M-PCNL	S-PCNL	M-PCNL	S-PCNL	M-PCNL	S-PCNL	M-PCNL	S-PCNL		M-PCNL	S-PCNL	M-PCNL	S-PCNL	M-PCNL	S-PCNL	M-PCNL	S-PCNL		
Cheng et al, 2010^16^	NI*	NI*	NI	NI	NI*	NI*	NI*	NI*	Prone	16 Fr	24 F	8/9.8 Fr	20.8 Fr	FD	FD	Pneumatic	Ultrasound+Pneumatic	No residual stone fragments ≥4 mm	X-ray (KUB) and abdominal USG week 1
Zhong et al, 2011^17^	41.00 (26-76)	38.00 (26-64)	NI	NI	14/15	11/14	11.7 (8.8-22.8) cm^2^	10.8 (8.4-20.2) cm^2^	Prone	16 Fr	26 Fr	8/9.8 Fr	NI	FD	FD	Pneumatic	Pneumatic	No symptomatic, obstructing, infected residual stone fragments ≥4 mm	X-ray (KUB) day 1
Wu et al, 2017^18^	NI*	NI*	NI*	NI*	NI*	NI*	NI*	NI*	Prone	16 Fr	24 F	8/9.8 Fr	20.8 Fr	FD	FD	Ultrasound	Ultrasound	No residual stone fragments ≥4 mm	X-ray (KUB) day 1
Du et al, 2018^19^	41.2 ± 16.9 years	44.5 ± 18.7 years	NI	NI	181/123	179/118	37.28 ± 21.6 cm^2^	35.21 ± 25.29 cm^2^	Prone	16-18 Fr	24 Fr	12 Fr	NI	FD	FD	Laser	Laser	No residual stone fragments >4 mm	X-ray (KUB) day 3-5, CT-scan (as necessary) to confirm residual stone fragments
Khadgi et al, 2021^20^	43.7 ± 13.9 years	51.9 ± 9.7 years	29 ± 3.3 kg/m^2^	34 ± 6 kg/m^2^	44/39	32/28	Guy’s stone score III-IV	Guy’s stone score III-IV	Prone	18-20 Fr	30 Fr	12 Fr	24 Fr	SSD	AMD/BD	Pneumatic	Pneumatic	No infected residual stone fragments ≥4 mm	X-ray (KUB) and NCCT-scan before discharge
Guliev et al, 2022^21^	51.0 ± 10.5 years	48.5 ± 15.0 years	NI	NI	17/15	31/27	NI	NI	Prone	18-20 Fr	28-30 Fr	15 Fr	24 Fr	SSD	SSD	Laser	Ultrasound	No residual stone fragments ≥4 mm	X-ray (KUB) and CT-scan before discharge
Khan et al, 2023^22^	37.8 ± 8.78 years	37.04 ± 4.04 years	24.1 ± 5.62 kg/m^2^	23.4 ± 5.13 kg/m^2^	37/9	40/11	36.32 ± 3.1 mm	37.33 ± 4.09 mm	Prone or supine	18 Fr	28-30 Fr	12 Fr	24 Fr	SSD	SSD	Laser	Pneumatic	Complete clearance (no stone fragments seen in follow up)	X-ray (KUB) week 4, NCCT-scan (as necessary in week 4) to confirm residual stone fragments
Shen et al, 2023^23^	54.50 ± 11.22 years	55.46 ± 11.61 years	26.29 ± 3.98 kg/m^2^	26.26 ± 3.86 kg/m^2^	122/90	44/54	970.68 ± 539.35 mm^2^	1632.86 ± 751.79 mm^2^	Prone	16/18 Fr	24 Fr	NI	NI	FD	FD	Laser	Ultrasound	No residual stone fragments ≥3 mm	CT-scan day 3
Sultan et al, 2023^24^	51.2 years (range: 18-85 years)		NI	NI	64/11	68/7	4.7 cm (range: 2-8 cm)		Prone	18 Fr	28 Fr	16 Fr	24 Fr	NI	NI	Pneumatic	Pneumatic	Complete clearance (no stone fragments seen in follow up)	X-ray (KUB) and NCCT-scan before discharge
Nawaz et al, 2024^25^	NI	NI	NI	NI	62/31	47/22	26.8 ± 5.8 mm	32.3 ± 10.3 mm	Supine	18 Fr	28 Fr	16 Fr	NI	PD	MD	Pneumatic	Pneumatic	No residual stone fragments >4 mm	X-ray (KUB) month 1 and abdominal USG month 1

AMD, Alken metal dilator; BD, balloon dilator; BMI, body mass index; FD, fascial dilator; F, Female; M, Male; NCCT scan, non-contrast CT scan. NI, no information. NI*, no information on patients with staghorn calculi; PD, plastic dilator; SFR, stone free rate; SSD, single step dilation.

**Supplementary Table 1.** Keywords used in each database

Database	Keywords	
PubMed	(“staghorn calculi” [MeSH Terms] OR “staghorn calculi” [Title/Abstract] OR “staghorn stone” [Title/Abstract]) AND (“nephrolithotomy, percutaneous” [MeSH Terms] OR “percutaneous nephrolithotomy” [Title/Abstract] OR “mini percutaneous nephrolithotomy” [Title/Abstract] OR “minimally invasive percutaneous nephrolithotomy” [Title/Abstract] OR “miniaturized percutaneous nephrolithotomy” [Title/Abstract] OR “mpcnl” [Title/Abstract] OR “m pcnl” [Title/Abstract] OR “mini perc” [Title/Abstract] OR “pcnl” [Title/Abstract])	
Scopus	TITLE-ABS-KEY (“staghorn calculi” OR “staghorn stone”) AND TITLE-ABS-KEY (“nephrolithotomy, percutaneous” OR “percutaneous nephrolithotomy” OR “mini percutaneous nephrolithotomy” OR “minimally invasive percutaneous nephrolithotomy” OR “miniaturized percutaneous nephrolithotomy” OR “mpcnl” OR “m pcnl” OR “mini perc” OR “pcnl”)	
ProQuest	noft (staghorn calculi OR staghorn stone) AND noft (nephrolithotomy, percutaneous OR percutaneous nephrolithotomy OR mini percutaneous nephrolithotomy OR minimally invasive percutaneous nephrolithotomy OR miniaturized percutaneous nephrolithotomy OR mpcnl OR m pcnl OR mini perc OR pcnl)	
Cochrane Central Register of Controlled Trials (CENTRAL)	#1	MeSH descriptor: [Staghorn Calculi] explode all trees
	#2	(staghorn calculi OR staghorn stone):ti,ab,kw
	#3	#1 OR #2
	#4	MeSH descriptor: [Nephrolithotomy, Percutaneous] explode all trees
	#5	percutaneous nephrolithotomy OR mini percutaneous nephrolithotomy OR minimally invasive percutaneous nephrolithotomy OR miniaturized percutaneous nephrolithotomy OR mpcnl OR m pcnl OR mini perc OR pcnl):ti,ab,kw
	#6	#4 OR #5
	#7	#3 AND #6
ClinicalTrials.gov	Condition/disease	staghorn calculi OR staghorn stone
	Intervention/treatment	percutaneous nephrolithotomy OR mini percutaneous nephrolithotomy OR minimally invasive percutaneous nephrolithotomy OR miniaturized percutaneous nephrolithotomy OR m pcnl OR mini perc OR pcnl
Google Scholar	with all of the words	“mini percutaneous nephrolithotomy” OR “pcnl” OR “mini pcnl” OR “staghorn calculi”
	with the exact phrase	percutaneous nephrolithotomy
	with at least one of the words	“staghorn calculi” OR pcnl
	without the words	-
	where my words occur	anywhere in the article

**Supplementary Table 2.** Sensitivity analysis of SFR

Excluded Study	Pooled OR	95% CI		Cochran Q	*P*	I² (%)
		Low	High			
Cheng et al 2010^16^	1.15	0.91	1.45	15.96	.04	50
Zhong et al 2011^17^	1.08	0.86	1.37	12.35	.14	35
Wu et al 2017^18^	1.14	0.90	1.45	16.08	.04	50
Du et al 2018^19^	1.20	0.89	1.61	15.98	.04	50
Khadgi et al 2021^20^	1.18	0.93	1.50	14.68	.07	45
Guliev et al 2022^21^	1.15	0.91	1.45	15.98	.04	50
Khan et al 2023^22^	1.08	0.85	1.37	12.07	.15	34
Shen et al 2023^23^	1.16	0.9	1.51	16.06	.04	50
Sultan et al 2023^24^	1.18	0.93	1.49	13.06	.11	39
Nawaz et al 2024^25^	1.06	0.83	1.35	12.48	.13	36

**Supplementary Table 3.** Sensitivity analysis of single tract access

Excluded Study	Pooled OR	95% CI		Cochran Q	*P*	I² (%)
		Low	High			
Zhong et al 2011^17^	1.78	0.57	5.57	36.37	< .001	92
Du et al 2018^19^	1.04	0.22	4.86	29.25	< .001	87
Khadgi et al 2021^20^	0.60	0.15	2.44	32.01	< .001	91
Guliev et al 2022^21^	0.70	0.14	3.47	51.08	< .001	94
Nawaz et al 2024^25^	0.63	0.13	3.02	46.01	< .001	94

**Supplementary Table 4.** Sensitivity analysis of operative time

Excluded Study	Pooled MD	95% CI		Cochran Q	*P*	I² (%)
		Low	High			
Cheng et al 2010^16^	13.84	5.06	22.62	64.50	< .001	92
Zhong et al 2011^17^	13.45	2.63	24.27	64.42	< .001	95
Wu et al 2017^18^	14.71	5.63	23.80	60.26	< .001	92
Du et al 2018^19^	11.60	3.31	19.90	45.60	< .001	89
Khadgi et al 2021^20^	17.61	10.67	24.54	38.63	< .001	87
Khan et al 2023^22^	15.26	6.51	24.01	55.66	< .001	91
Shen et al 2023^23^	11.66	3.17	20.16	50.63	< .001	90

**Supplementary Table 5.** Sensitivity analysis of blood transfusion

Excluded Study	Pooled OR	95% CI		Cochran Q	*P*	I² (%)
		Low	High			
Zhong et al 2011^17^	0.47	0.30	0.74	4.33	.50	0
Du et al 2018^19^	0.33	0.18	0.59	1.31	.93	0
Khadgi et al 2021^20^	0.52	0.32	0.83	2.71	.74	0
Guliev et al 2022^21^	0.47	0.30	0.75	4.34	.50	0
Khan et al 2023^22^	0.47	0.28	0.77	4.55	.47	0
Shen et al 2023^23^	0.47	0.30	0.74	4.22	.52	0
Sultan et al 2023^24^	0.47	0.29	0.77	4.53	.48	0

**Supplementary Table 6.** Sensitivity analysis of hemoglobin drop

Excluded Study	Pooled MD	95% CI		Cochran Q	*P*	I² (%)
		Low	High			
Zhong et al 2011^17^	-0.31	-0.89	0.27	2.86	.09	65
Khan et al 2023^22^	-0.29	-0.72	0.13	3.97	.17	75
Nawaz et al 2024^25^	-0.53	-0.78	-0.28	0.25	.62	0

**Supplementary Table 7.** Sensitivity analysis of postoperative fever

Excluded Study	Pooled OR	95% CI		Cochran Q	*P*	I² (%)
		Low	High			
Zhong et al 2011^17^	1.17	0.8	1.71	6.47	.16	38
Du et al 2018^19^	0.72	0.42	1.26	1.77	.77	0
Khadgi et al 2021^20^	1.14	0.78	1.66	6.5	.16	38
Guliev et al 2022^21^	1.15	0.79	1.68	6.59	.16	39
Shen et al 2023^23^	1.38	0.91	2.1	3.01	.55	0
Sultan et al 2023^24^	1.24	0.84	1.82	5.05	.28	21

**Supplementary Table 8.** Sensitivity analysis overall postoperative complications

Excluded Study	Pooled OR	95% CI		Cochran Q	*P*	I² (%)
		Low	High			
Zhong et al 2011^17^	0.64	0.26	1.55	20.72	< .001	81
Khadgi et al 2021^20^	0.68	0.27	1.71	19.74	< .001	80
Guliev et al 2022^21^	0.63	0.26	1.56	20.75	< .001	80
Khan et al 2023^22^	0.45	0.3	0.66	1.75	.78	0
Shen et al 2023^23^	0.66	0.24	1.8	19.12	< .001	79
Sultan et al 2023^24^	0.75	0.33	1.71	17.34	.001	77

**Supplementary Table 9.** Sensitivity analysis of hospital stay

Excluded Study	Pooled OR	95% CI		Cochran Q	*P*	I² (%)
		Low	High			
Zhong et al 2011^17^	-2.05	-3.87	-0.23	70.00	< .001	97
Khadgi et al 2021^20^	-0.47	-2.39	1.46	41.20	< .001	95
Shen et al 2023^23^	-0.64	-2.83	1.54	110.37	< .001	98
Nawaz et al 2024^25^	-1.29	-4.32	1.73	89.76	< .001	98

## Data Availability

The data of this study is available upon request to the corresponding author
