# Supplementary material for: Comparison on Surgical Outcomes of Mini-Versus Standard-Percutaneous Nephrolithotomy in Staghorn Calculi: A Systematic Review and Meta-Analysis
Source: Urol Res Pract. 2025 Mar 6;50(5):281–90. doi: 10.5152/tud.2025.24125 (PMC11923600; doi:10.5152/tud.2025.24125)
Supplement: Supplementary Material [file supplementary_material.pdf]

Supplementary Table 1. Keywords used in each database

Database	Keywords	
PubMed	(“staghorn calculi” [MeSH Terms] OR “staghorn calculi” [Title/Abstract] OR “staghorn stone” [Title/Abstract]) AND (“nephrolithotomy, percutaneous” [MeSH Terms] OR “percutaneous nephrolithotomy” [Title/Abstract] OR “mini percutaneous nephrolithotomy” [Title/Abstract] OR “minimally invasive percutaneous nephrolithotomy” [Title/Abstract] OR “miniaturized percutaneous nephrolithotomy” [Title/Abstract] OR “mpcnl” [Title/Abstract] OR “m pcnl” [Title/Abstract] OR “mini perc” [Title/Abstract] OR “pcnl” [Title/Abstract])	
Scopus	TITLE-ABS-KEY (“staghorn calculi” OR “staghorn stone”) AND TITLE-ABS-KEY (“nephrolithotomy, percutaneous” OR “percutaneous nephrolithotomy” OR “mini percutaneous nephrolithotomy” OR “minimally invasive percutaneous nephrolithotomy” OR “miniaturized percutaneous nephrolithotomy” OR “mpcnl” OR “m pcnl” OR “mini perc” OR “pcnl”)	
ProQuest	noft (staghorn calculi OR staghorn stone) AND noft (nephrolithotomy, percutaneous OR percutaneous nephrolithotomy OR mini percutaneous nephrolithotomy OR minimally invasive percutaneous nephrolithotomy OR miniaturized percutaneous nephrolithotomy OR mpcnl OR m pcnl OR mini perc OR pcnl)	
Cochrane Central Register of Controlled Trials (CENTRAL)	#1	MeSH descriptor: [Staghorn Calculi] explode all trees
	#2	(staghorn calculi OR staghorn stone):ti,ab,kw
	#3	#1 OR #2
	#4	MeSH descriptor: [Nephrolithotomy, Percutaneous] explode all trees
	#5	percutaneous nephrolithotomy OR mini percutaneous nephrolithotomy OR minimally invasive percutaneous nephrolithotomy OR miniaturized percutaneous nephrolithotomy OR mpcnl OR m pcnl OR mini perc OR pcnl):ti,ab,kw
	#6	#4 OR #5
	#7	#3 AND #6
ClinicalTrials.gov	Condition/disease	staghorn calculi OR staghorn stone
	Intervention/treatment	percutaneous nephrolithotomy OR mini percutaneous nephrolithotomy OR minimally invasive percutaneous nephrolithotomy OR miniaturized percutaneous nephrolithotomy OR m pcnl OR mini perc OR pcnl
Google Scholar	with all of the words	“mini percutaneous nephrolithotomy” OR “pcnl” OR “mini pcnl” OR “staghorn calculi”
	with the exact phrase	percutaneous nephrolithotomy
	with at least one of the words	“staghorn calculi” OR pcnl
	without the words	-
	where my words occur	anywhere in the article

Supplementary Table 2. Sensitivity analysis of SFR

		95% CI				
**Excluded Study**	**Pooled OR**	**Low**	**High**	**Cochran Q**	***P***	**I^2 (%)**
Cheng et al 2010[16]	1.15	0.91	1.45	15.96	.04	50
Zhong et al 2011[17]	1.08	0.86	1.37	12.35	.14	35
Wu et al 2017[18]	1.14	0.90	1.45	16.08	.04	50
Du et al 2018[19]	1.20	0.89	1.61	15.98	.04	50
Khadgi et al 2021[20]	1.18	0.93	1.50	14.68	.07	45
Guliev et al 2022[21]	1.15	0.91	1.45	15.98	.04	50
Khan et al 2023[22]	1.08	0.85	1.37	12.07	.15	34
Shen et al 2023[23]	1.16	0.9	1.51	16.06	.04	50
Sultan et al 2023[24]	1.18	0.93	1.49	13.06	.11	39
Nawaz et al 2024[25]	1.06	0.83	1.35	12.48	.13	36

Supplementary Table 3. Sensitivity analysis of single tract access

		95% CI				
**Excluded Study**	**Pooled OR**	**Low**	**High**	**Cochran Q**	***P***	**I^2 (%)**
Zhong et al 2011[17]	1.78	0.57	5.57	36.37	< .001	92
Du et al 2018[19]	1.04	0.22	4.86	29.25	< .001	87
Khadgi et al 2021[20]	0.60	0.15	2.44	32.01	< .001	91
Guliev et al 2022[21]	0.70	0.14	3.47	51.08	< .001	94
Nawaz et al 2024[25]	0.63	0.13	3.02	46.01	< .001	94

Supplementary Table 4. Sensitivity analysis of operative time

Excluded Study	Pooled MD	95% CI Low	95% CI High	Cochran Q	*P*	I^2 (%)
Cheng et al 2010[16]	13.84	5.06	22.62	64.50	< .001	92
Zhong et al 2011[17]	13.45	2.63	24.27	64.42	< .001	95
Wu et al 2017[18]	14.71	5.63	23.80	60.26	< .001	92
Du et al 2018[19]	11.60	3.31	19.90	45.60	< .001	89
Khadgi et al 2021[20]	17.61	10.67	24.54	38.63	< .001	87
Khan et al 2023[22]	15.26	6.51	24.01	55.66	< .001	91
Shen et al 2023[23]	11.66	3.17	20.16	50.63	< .001	90

Supplementary Table 5. Sensitivity analysis of blood transfusion

Excluded Study	Pooled OR	95% CI Low	95% CI High	Cochran Q	*P*	I^2 (%)
Zhong et al 2011[17]	0.47	0.30	0.74	4.33	.50	0
Du et al 2018[19]	0.33	0.18	0.59	1.31	.93	0
Khadgi et al 2021[20]	0.52	0.32	0.83	2.71	.74	0
Guliev et al 2022[21]	0.47	0.30	0.75	4.34	.50	0
Khan et al 2023[22]	0.47	0.28	0.77	4.55	.47	0
Shen et al 2023[23]	0.47	0.30	0.74	4.22	.52	0
Sultan et al 2023[24]	0.47	0.29	0.77	4.53	.48	0

Supplementary Table 6. Sensitivity analysis of hemoglobin drop

Excluded Study	Pooled MD	95% CI Low	95% CI High	Cochran Q	*P*	I^2 (%)
Zhong et al 2011[17]	-0.31	-0.89	0.27	2.86	.09	65
Khan et al 2023[22]	-0.29	-0.72	0.13	3.97	.17	75
Nawaz et al 2024[25]	-0.53	-0.78	-0.28	0.25	.62	0

Supplementary Table 7. Sensitivity analysis of postoperative fever

Excluded Study	Pooled OR	95% CI Low	95% CI High	Cochran Q	*P*	I^2 (%)
Zhong et al 2011[17]	1.17	0.8	1.71	6.47	.16	38
Du et al 2018[19]	0.72	0.42	1.26	1.77	.77	0
Khadgi et al 2021[20]	1.14	0.78	1.66	6.5	.16	38
Guliev et al 2022[21]	1.15	0.79	1.68	6.59	.16	39
Shen et al 2023[23]	1.38	0.91	2.1	3.01	.55	0
Sultan et al 2023[24]	1.24	0.84	1.82	5.05	.28	21

Supplementary Table 8. Sensitivity analysis overall postoperative complications

Excluded Study	Pooled OR	95% CI Low	95% CI High	Cochran Q	*P*	I^2 (%)
Zhong et al 2011[17]	0.64	0.26	1.55	20.72	< .001	81
Khadgi et al 2021[20]	0.68	0.27	1.71	19.74	< .001	80
Guliev et al 2022[21]	0.63	0.26	1.56	20.75	< .001	80
Khan et al 2023[22]	0.45	0.3	0.66	1.75	.78	0
Shen et al 2023[23]	0.66	0.24	1.8	19.12	< .001	79
Sultan et al 2023[24]	0.75	0.33	1.71	17.34	.001	77

Supplementary Table 9. Sensitivity analysis of hospital stay

Excluded Study	Pooled OR	95% CI Low	95% CI High	Cochran Q	*P*	I^2 (%)
Zhong et al 2011[17]	-2.05	-3.87	-0.23	70.00	< .001	97
Khadgi et al 2021[20]	-0.47	-2.39	1.46	41.20	< .001	95
Shen et al 2023[23]	-0.64	-2.83	1.54	110.37	< .001	98
Nawaz et al 2024[25]	-1.29	-4.32	1.73	89.76	< .001	98

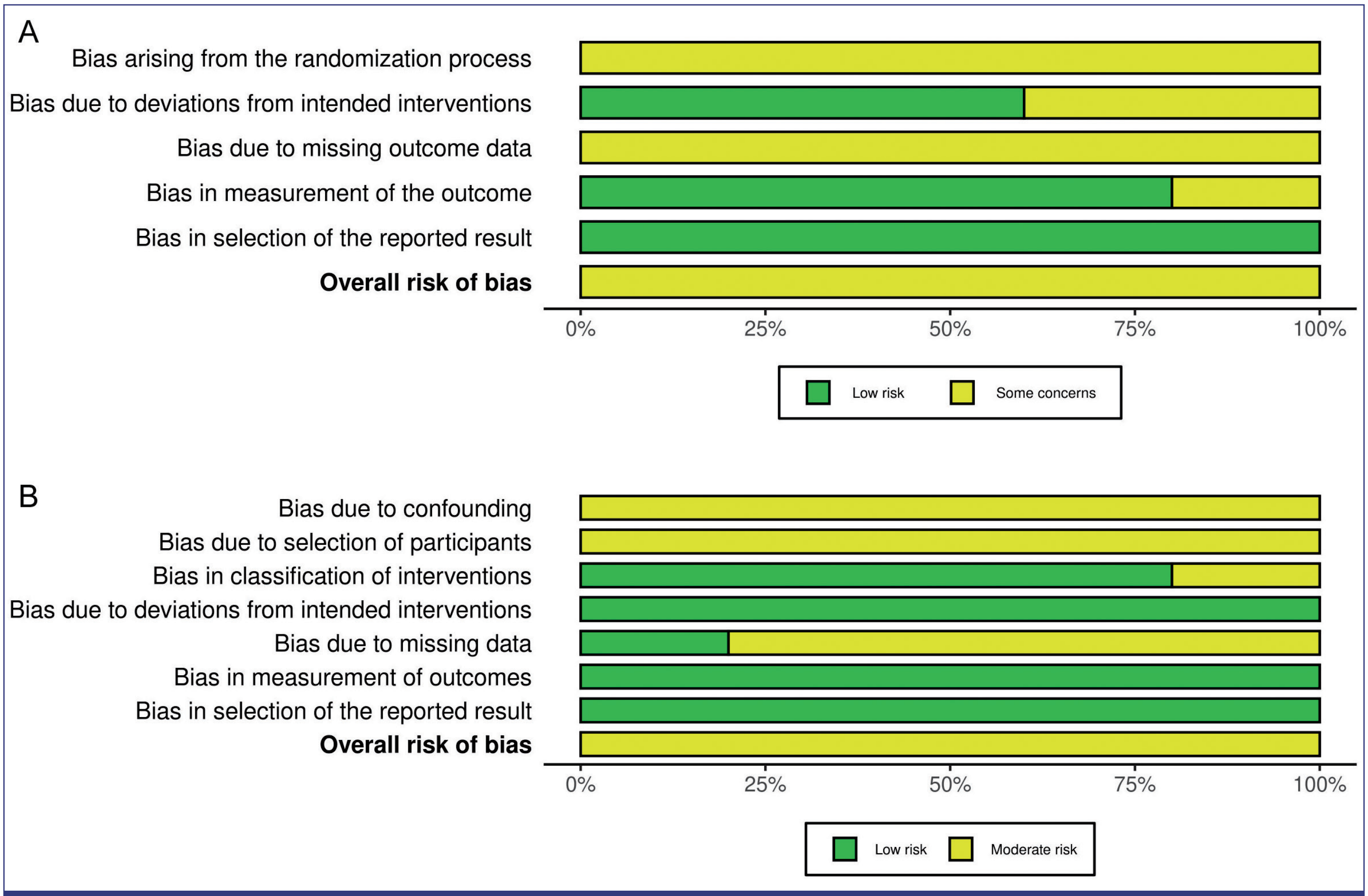

Supplementary Figure 1. a, b. Risk of bias assessment of included studies. (a) RoB 2.0 for RCT studies, (b) ROBINS-I for included cohort studies.

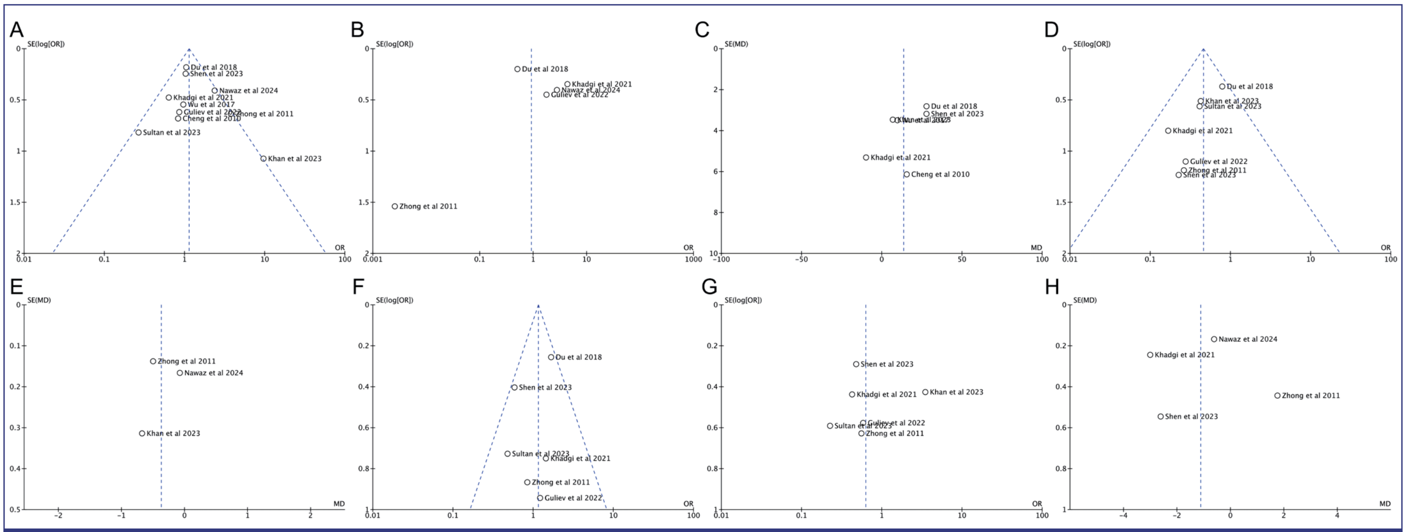

Supplementary Figure 2. a-h. Funnel plot of meta-analysis reporting surgical outcomes and postoperative complications. (a) SFR, (b) single tract access, (c) operative time, (d) blood transfusion, (e) hemoglobin drop, (f) postoperative fever, (g) overall postoperative complications, (h) hospital stay.
